# Determination of Exploratory Metastasis Biomarkers in Cancer of Unknown Primary Using Metastatic Colon Cancer as a Model: RNA Sequencing Bioinformatics Analysis

**DOI:** 10.3390/cimb48101058

**Published:** 2026-10-14

**Authors:** Rawikant Kamolphiwong, Pongsakorn Choochuen, Maliwan Songserm, Patcharaporn Thongwatchara, Arunee Dechaphunkul, Chirawadee Sathitruangsak, Jirapat Wonglhow, Patrapim Sunpaweravong

**Affiliations:** 1 Division of Medical Oncology, Department of Internal Medicine, Faculty of Medicine, Prince of Songkla University, Songkhla 90110, Thailand; k.rawikant@gmail.com (R.K.); maliwan_99@hotmail.com (M.S.); phatch.th@hotmail.com (P.T.); dr.arunee@gmail.com (A.D.); sjirawadee@gmail.com (C.S.); jirapat.jw@gmail.com (J.W.); 2 Department of Biomedical Sciences and Biomedical Engineering, Faculty of Medicine, Prince of Songkla University, Songkhla 90110, Thailand; pongsakorn.choochuen@gmail.com

**Keywords:** cancer of unknown primary, colon cancer, RNA sequencing, The Cancer Genome Atlas, organ origin, bioinformatics

## Abstract

We aimed to explore the real-world practice setting and identify common genetic signaling or patterns in cancer of unknown primary (CUP) using colon cancer as a model through RNA sequencing bioinformatics analysis. Data were retrieved and analyzed using the hospital information system. We compared the number of CUP patients involved in Medical Oncology services with those who were not, and used a bioinformatics approach to analyze RNA sequencing datasets from The Cancer Genome Atlas for metastatic colon cancer as a prototype and compare information between the primary tumor and its metastatic sites. Comparisons for identifying differentially expressed genes included liver metastases versus primary tumors, lymph node metastases versus primary tumors, and all colon tumors with metastasis versus those without. Over 20 years, the hospital identified 1193 CUP patients; 154 received Medical Oncology services. We sourced 37 primary and 9 metastatic (5 liver, 4 lymph node) colon cancer RNA sequencing datasets for bioinformatics analysis. Multigroup analysis identified *ROPN1*, *Y_RNA*, and *OR4F4* as highly expressed in both metastatic types. High *ROPN1* expression was associated with poor overall and disease-free survival rates. RNA sequencing bioinformatics is potentially useful for identifying exploratory metastasis biomarkers in CUP. *ROPN1* may be linked to metastasis and unfavorable prognosis in colon cancer. Further analysis across paired primary/metastatic panels from multiple cancer types is warranted to determine the lineage-tracing utility in CUP.

## 1. Introduction

Cancer of unknown primary (CUP) is the term used to identify patients with heterogeneous metastatic disease, the primary site of which cannot be determined. The characteristic features of CUP include early metastatic dissemination, aggressiveness, poor prognosis/response, and short life expectancy [1,2]. CUP accounts for 3–5% of all cancers diagnosed worldwide and is among the top 10 most common causes of cancer-related deaths [2,3]. It can be divided into two subgroups depending on the treatment outcomes: favorable and unfavorable, with response rates of 80–85% and 15–20%, respectively. Patient outcomes and disease control in cancers with metastasis, such as breast, ovarian, lung, colorectal, and malignant melanoma, have improved owing to new classes of chemotherapy and biological agents. Therefore, identifying the origin of CUP is important for more accurate treatment. Recently, omics studies have been used to identify the tissue origin and exploratory metastasis biomarkers for treatment [1]. However, if any clues from clinical, imaging, and laboratory evaluations are unambiguous, site-specific treatment might be better than empirical chemotherapy [4].

The Cancer Genome Atlas (TCGA) was launched in 2005 with the main purpose of accelerating the comprehensive understanding of cancer genetics through innovative genomic analysis technologies and developing new diagnostic methods and cancer therapies [5]. Researchers can access and download TCGA data through the Genomic Data Commons Portal. TCGA includes 32 cancer types with primary untreated tumors and matched normal tissue, blood samples, and frozen resection samples. All samples contain at least 80% tumor nuclei (https://www.cancer.gov/about-nci/organization/ccg/research/structural-genomics/tcga (accessed on 6 November 2023)).

Bioinformatics is a powerful tool for analyzing gene expression and drug discovery by combining biological, mathematical, statistical, and computational methods to address specific goals [6]. RNA sequencing is a high-throughput method used to understand cellular transcriptome biology. Moreover, RNA sequencing enables researchers to discover gene expression patterns and signaling pathways and investigate novel genes for the diagnosis or treatment of a particular disease. Previous studies across various cancer types have used RNA sequencing to develop prognostic models and revealed that genes identified by RNA analysis, along with clinical data, form a strong predictive model [7]. This study aimed to explore the real-world practice setting of CUP and identify common genetic signaling or patterns in CUP using colon cancer as a model through RNA sequencing bioinformatics analysis.

## 2. Materials and Methods

We initially determined the proportion of patients diagnosed with CUP at PSU Hospital, a medical school-based setting in Southern Thailand. Patients with CUP receiving Medical Oncology services were identified to better understand real-world clinical practice. We then systematically retrieved published articles related to colon cancer with metastasis as a cancer prototype to examine the potential genes responsible for metastasis. The TCGA database was used to retrieve the RNA sequencing data to identify differentially expressed genes between the metastatic and primary cancer lesions. The retrieved genes from a previous publication using re-RNA analysis, along with selected potential genes, were compared to assess progression-free and overall survival in patients with colon cancer overexpressing these genes.

This study was conducted in accordance with the principles of the Declaration of Helsinki and approved by the Ethics Committee of the Faculty of Medicine, PSU (approval number REC 66-040-14-4). All study participants provided informed consent.

### 2.1. PSU Hospital Database Retrieval

We reviewed the PSU Hospital database obtained from Digital Innovation and Data Analytics to examine the number of patients with CUP treated by medical oncologists versus other specialties. Data from the years 2014–2022 were extracted. The search terms used to determine the number of patients with CUP were C800 Malignant neoplasms, unknown primary sites, and unspecified C809 Malignant neoplasms. These data may reflect the real-world clinical experience of patients with CUP treated by experts at our institution.

### 2.2. Systematic Review of Articles on Colon Cancer with Metastasis

We searched the Cochrane Library, PubMed (MEDLINE), and Embase databases for articles published in English for the last 10 years and up to January 2023, using the following search terms: colon cancer, metastasis, biomarker, expression, profiling, signature, sequencing, mutation, gene, genetic, genome, genomic, epigenomic, DNA, RNA, exome, transcriptome, methylome, protein, proteomics, multi-omics, methylation, epigenetic, ctDNA, cfDNA, and molecular. Duplicate data were removed, and abstracts of the retrieved articles were briefly screened to extract articles describing colon cancer and metastasis. Related articles were then screened to distinguish between original and non-original articles (e.g., review articles). Gene expression data comparing primary colon cancer and metastatic lesions were retrieved to identify the gene expression patterns.

### 2.3. Bioinformatics Analysis

#### 2.3.1. Data Collection and Transcriptome Data Processing

Data were processed using R (v4.3.2). Gene expression data for colorectal cancer tissues were obtained from the HCMI-CMDC project (https://portal.gdc.cancer.gov/projects/HCMI-CMDC (accessed on 6 November 2023)) using the TCGAbiolinks package (v2.28.3) [8,9,10], via the STAR–Counts workflow. The unstranded raw gene-level count matrix was retrieved and used as the input for all statistical analyses. FPKM values were generated from the same GDC data for exploratory visualization. Log-transformed FPKM values were used for principal component analysis of the transcriptome with the PCAtools package (v2.16.0). Differential gene expression analysis was performed on the unstranded raw counts using the edgeR package (v3.32.0) [11]. The same approach was used to compare the gene expression profiles between patients with different colorectal metastatic sites. A significance threshold of FDR ≤ 0.05 and |fold change (FC)| > 2 was used to determine statistical significance.

#### 2.3.2. Overall Survival and Disease-Free Survival

The genes of interest were analyzed using Gene Expression Profiling Interactive Analysis (GEPIA), a widely used web server for analyzing cancer and normal gene expression data, with downloaded TCGA and genotype-tissue expression data. We used the colon adenocarcinoma (COAD) dataset for this analysis, which was designed to resemble the CUP model [12]. A total of 275 colon cancer samples were available for this analysis. Patients were stratified into high- and low-expression groups using the median expression level (50% cutoff). Hazard ratios (HRs) and corresponding 95% confidence intervals (CIs) were estimated using the Cox proportional hazards model. The genes subjected to survival analysis were selected from the genes that were consistently identified across our preceding analyses.

## 3. Results

### 3.1. CUP from the Prince of Songkla University (PSU) Hospital Database

Between January 2002 and December 2022, 1193 patients with CUP were identified from the hospital information system by Digital Innovation and Data Analytics. We noted significantly fewer patients who received Medical Oncology services than those who did not (*p* < 0.001) (Table 1; Table S1).

### 3.2. Systematic Review of Articles on Colon Cancer with Metastasis

The database search identified 1022 published articles. Eighty-four articles were related to colon cancer and metastasis, and twenty-five of these were original articles. Among them, four articles describing gene expression were reviewed. We found only one article that compared metastatic and primary sites (Table S2). Therefore, the candidate genes from this study were retrieved for comparative analysis.

### 3.3. RNA Gene Expression Database Retrieved from TCGA

RNA sequencing data were retrieved from TCGA, along with metadata describing tissue type and organ origin. We collected RNA data from 37 primary sample sites of colon cancer and 9 metastatic sample sites (4 lymph nodes and 5 liver metastasis sites) (Table S3).

### 3.4. Identification of Differential Gene Expression (DGE)

We grouped the RNA data into three categories: (1) liver metastasis, (2) lymph node metastasis, and (3) metastasis versus non-metastasis. In the liver metastasis group, comparisons were made between the liver metastatic sites and primary colon cancer tissues. In the lymph node metastasis group, we compared metastatic lymph node sites and primary colon cancer tissues. Regarding the metastasis versus non-metastasis groups, we compared colon cancer tissue with clinical metastasis and colon cancer tissue without clinical metastasis.

**Figure 1:**
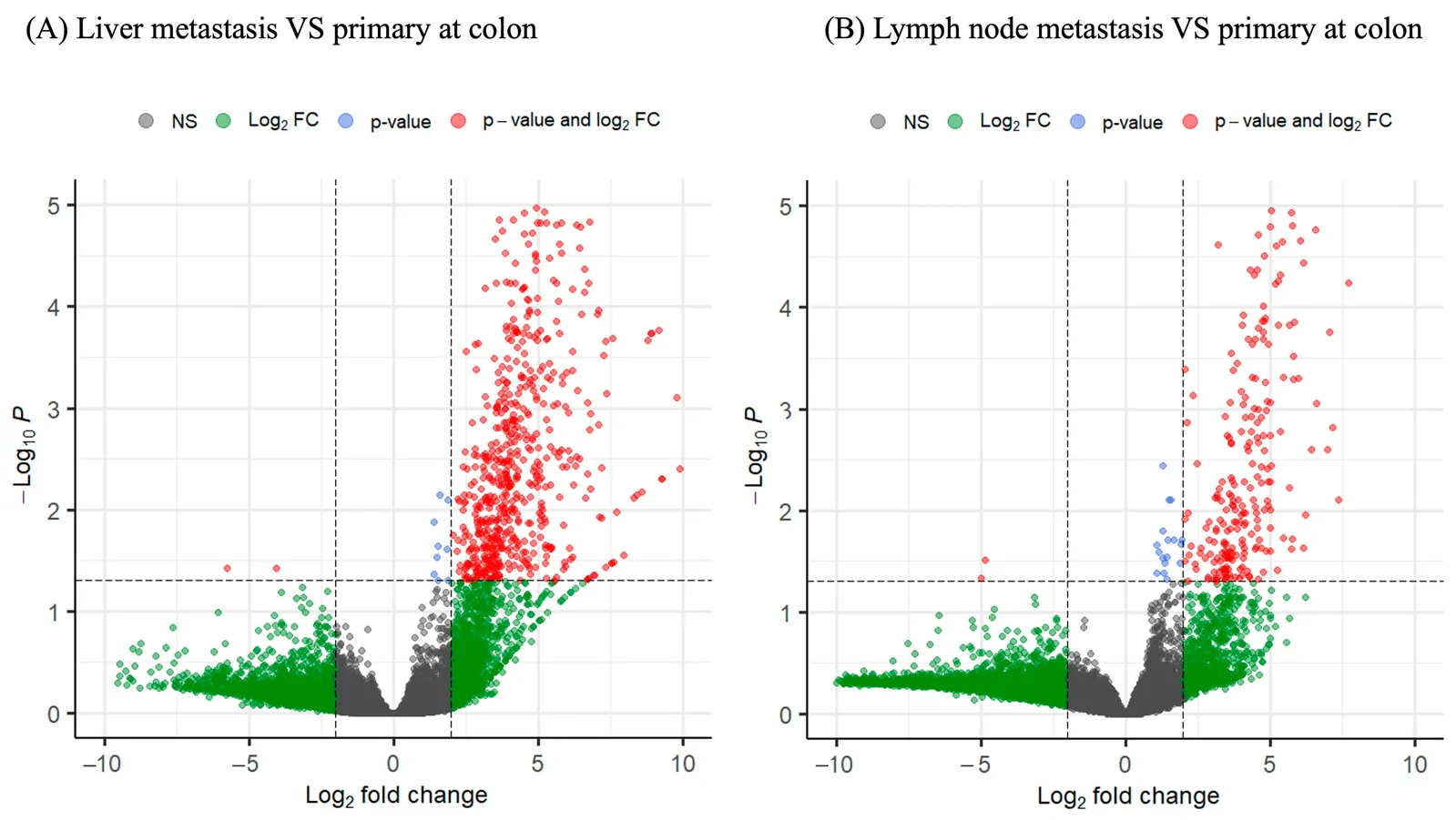
Volcano plot. (**A**) Differential gene expression of the liver metastasis tissue versus the primary colon tissue. (**B**) Differential gene expression of lymph node metastasis tissue versus the primary colon tissue. FC, fold change; log2FC, genes with log2 fold change ≥ 2; *p*-value, genes with *p*-value < 0.05; *p*-value and log2 FC, genes with *p*-value < 0.05 and log2 fold change ≥ 2; NS, not significant; VS, versus.

**Figure 2:**
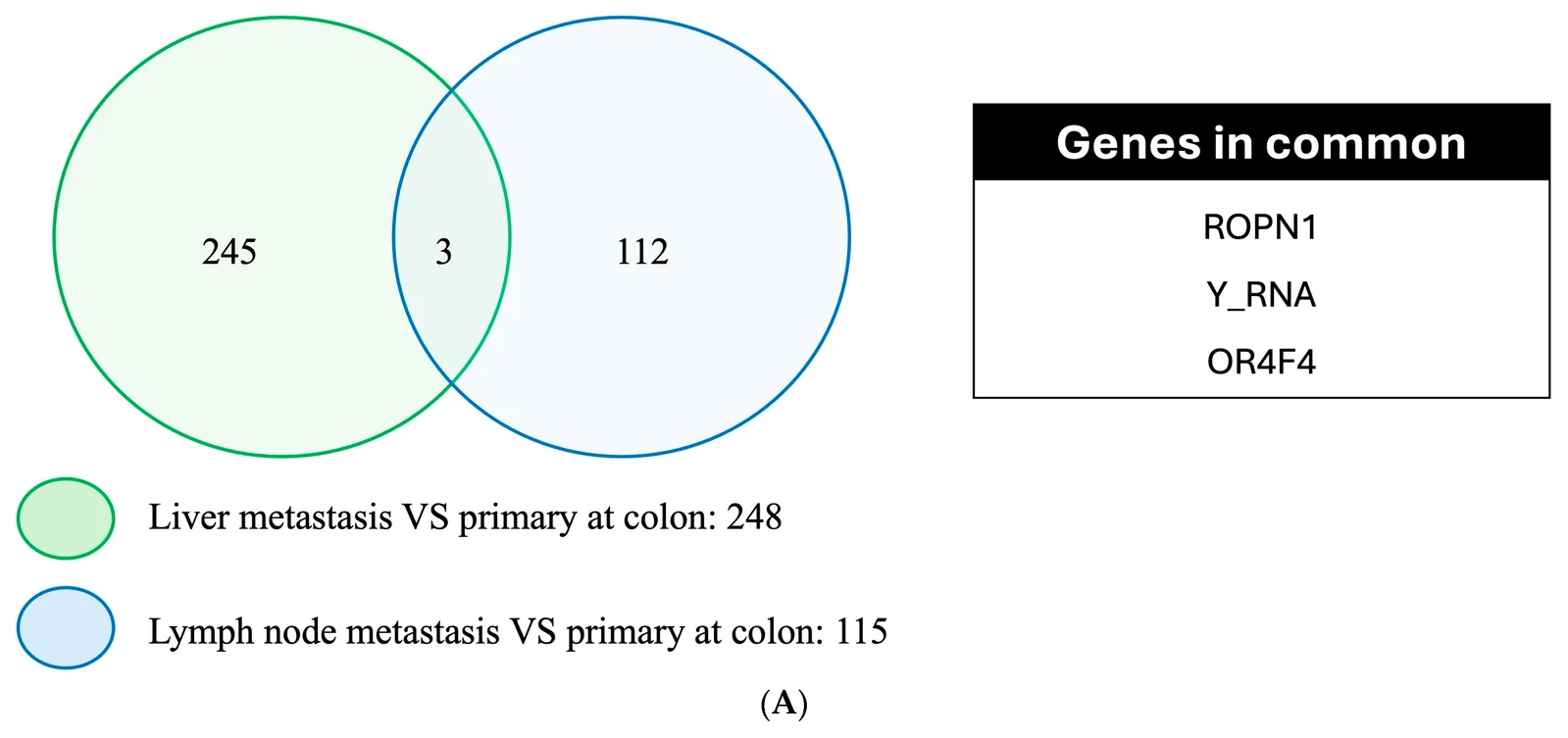

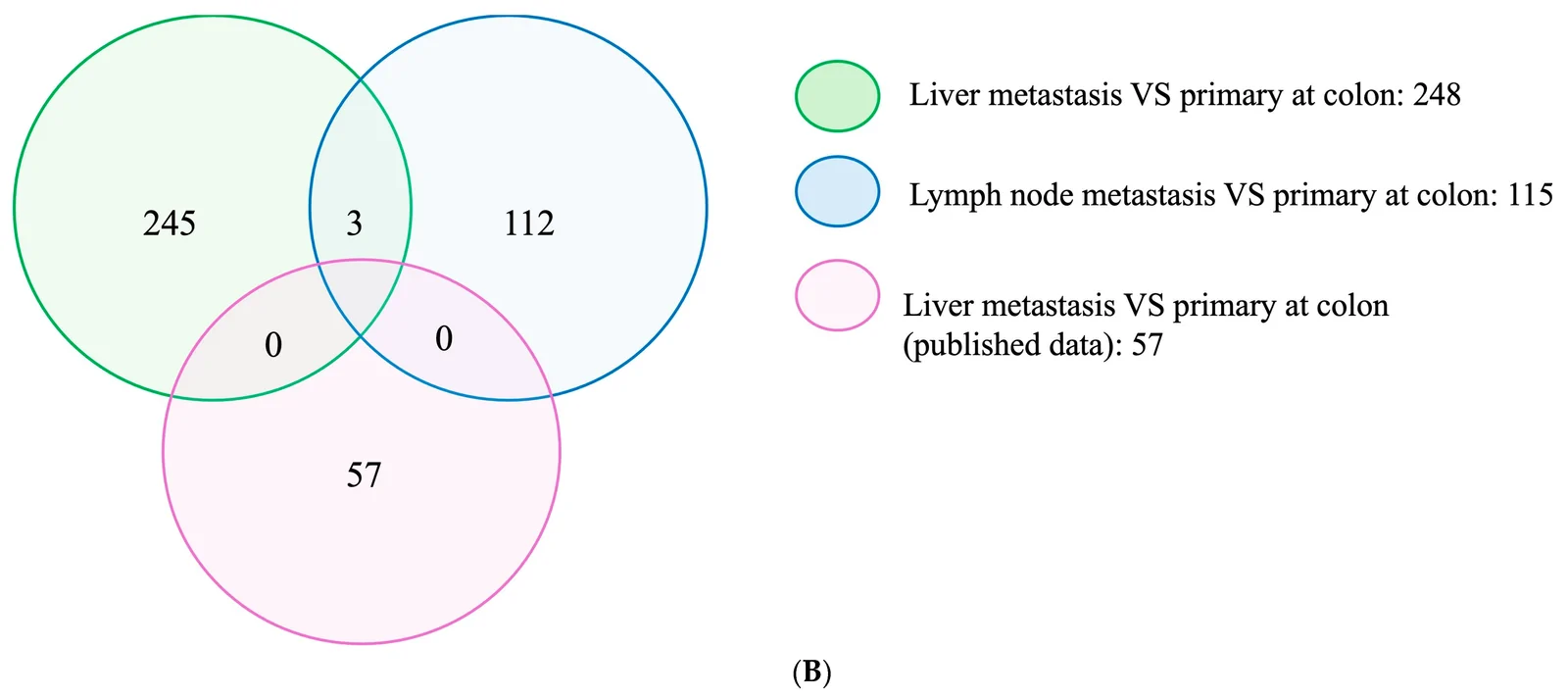
(**A**). Venn diagram of the two groups of genes of interest. VS, versus; *ROPN1*, ropporin-1; *Y_RNA*, cytoplasmic Y RNAs; *OR4F4*, olfactory receptor family 4 subfamily F member 4. (**B**). Venn diagram of the three groups of genes of interest.

There were 248 genes and 115 genes with log fold change ≥ 2 and adjusted *p*-value < 0.05 (false discovery rate [FDR]) in the liver metastasis group and the lymph node metastasis group, respectively. Only one gene, *FOXN4*, was downregulated (log fold change: −4.9, FDR: 0.03) in the liver metastasis group, and two genes, *HSPA8P14* (log fold change: −4.9, FDR: 0.02) and *RP4-758J24.4* (log fold change: −5.7, FDR: 0.046), were downregulated in the lymph node metastasis group. DGE is shown in Figure 1. Venn diagrams were created to identify the common upregulated genes shared by both groups. Three genes were identified (Figure 2A): Rhophilin-associated tail protein 1 (*ROPN1* or ropporin), *Y_RNA*, and *OR4F4*. However, we did not find any significant DGEs in the metastatic group versus the non-metastatic group.

**Figure 3:**
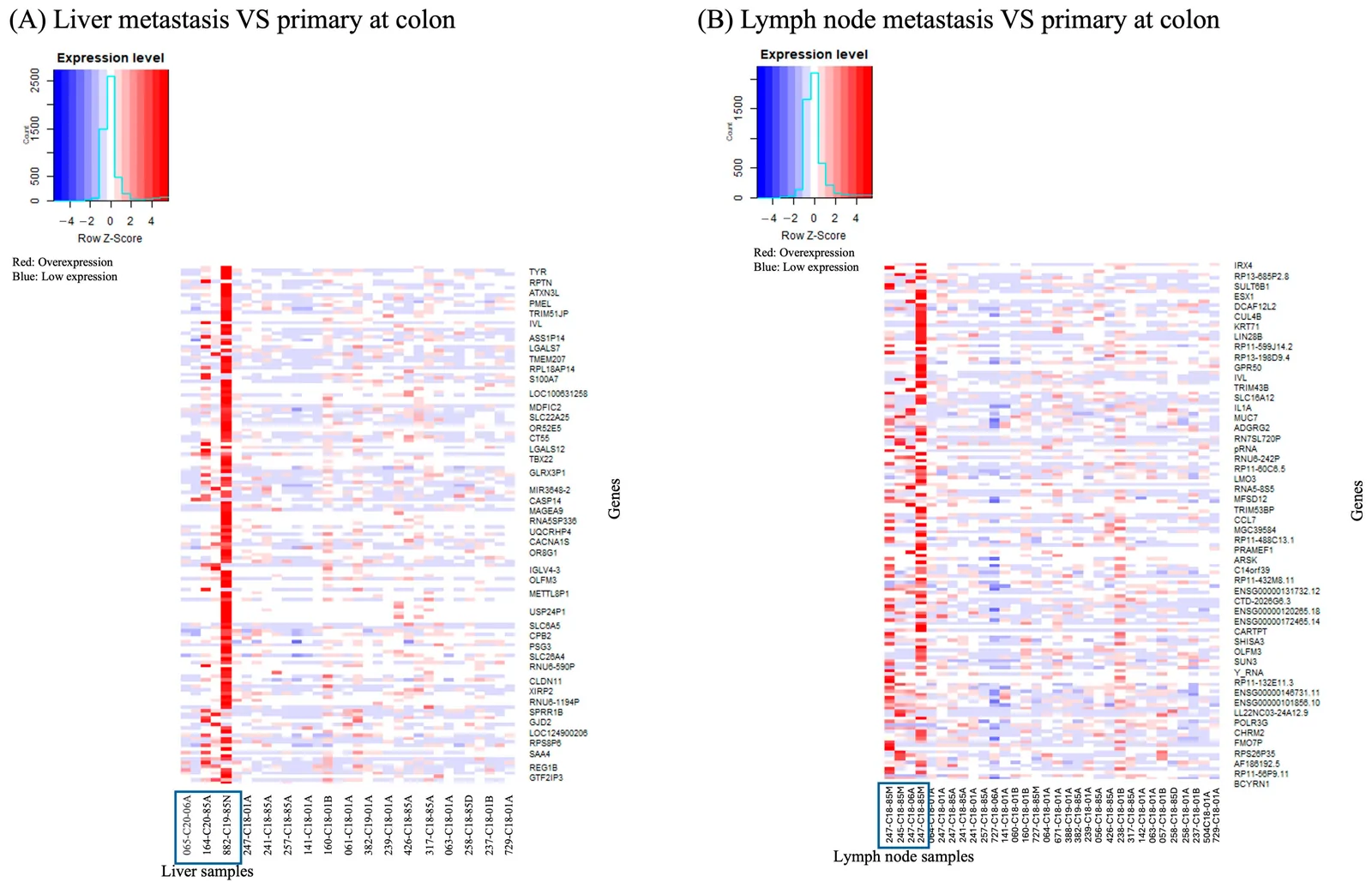
Heat map of gene expression between groups. (**A**) Liver metastasis and primary colon. (**B**) Lymph node metastasis and primary colon. VS, versus.

By retrieving articles from a systematic review of colon cancer metastasis, we identified candidate genes and compared them between those derived from the metastasis group and those from the primary colon cancer group. We then compared these genes with the DGE from the liver and lymph node metastasis groups. None of the input genes overlapped among the three groups (Figure 2B). Figure 3 shows a heatmap of gene expression in the liver and lymph node metastasis groups based on gene expression and samples. We found that metastatic samples showed upregulated gene expression.

**Figure 4:**
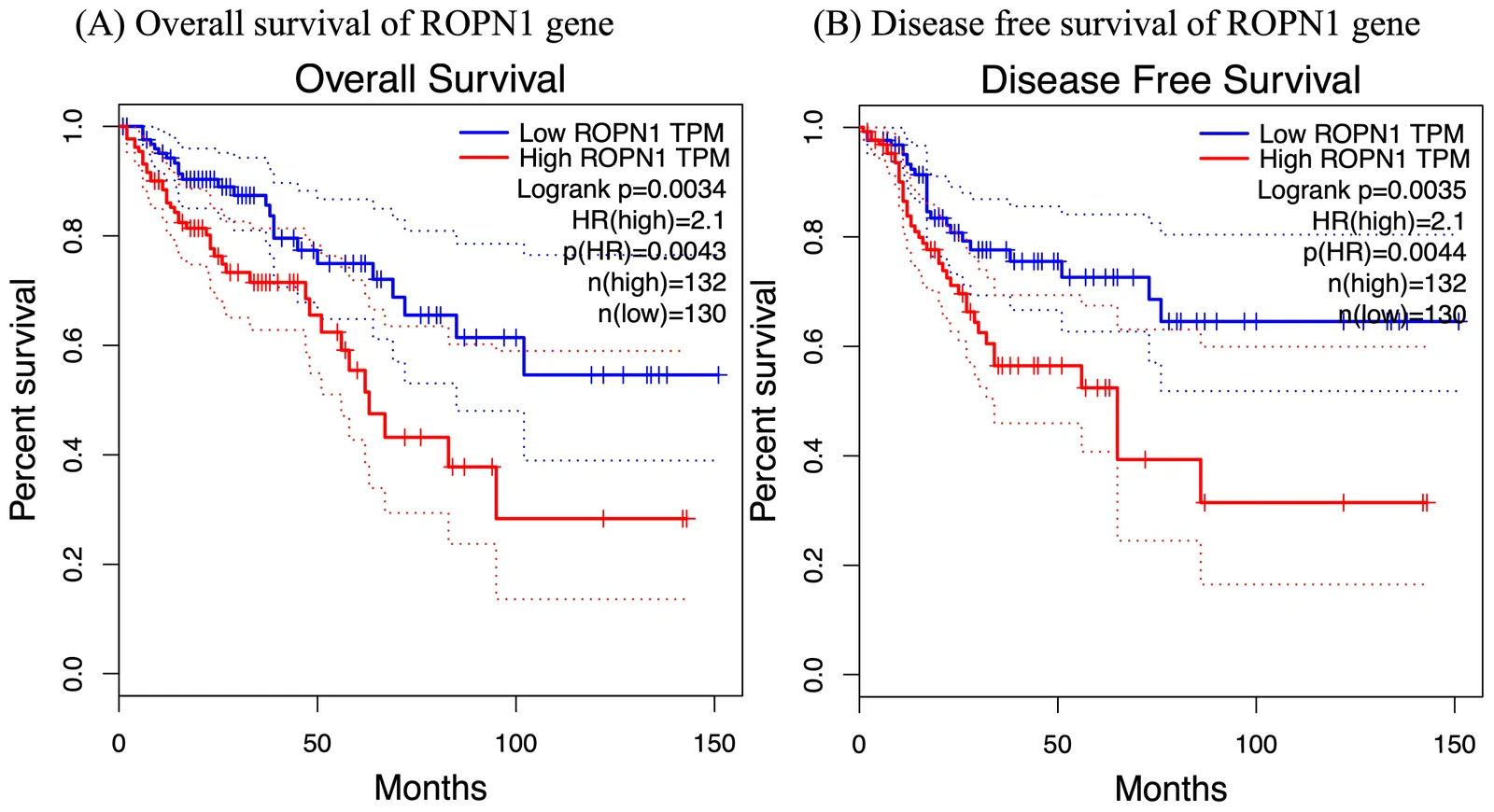
Overall and disease-free survival. (**A**) Overall survival and (**B**) disease-free survival of the *ROPN1* gene in colon cancer. *ROPN1*, ropporin-1; TPM, transcripts per million; HR, hazard ratio.

To evaluate the clinical significance of the three upregulated genes in the lymph node- and liver-metastatic groups, we analyzed overall and disease-free survival in patients expressing these genes. We found that high *ROPN1* expression was associated with decreased overall and disease-free survival (Figure 4).

## 4. Discussion

CUP is used to identify patients with heterogeneous metastatic disease, the primary site of which cannot be determined. The characteristic features of CUP include early metastatic dissemination, aggressiveness, poor prognosis/responses, and short life expectancy [1,2]. CUP accounts for 3–5% of all cancers diagnosed worldwide and is among the top ten most common causes of cancer-related deaths [2,3]. Common sites of metastasis include the liver, bones, lungs, and lymph nodes. Cancer cell clonal proliferation, invasion, and intravasation from the primary tumor, dissemination through circulation, colonization, and growth in different organs are the consequences of tumorigenesis. Two scenarios describe the pathophysiology of CUP. First, the parallel progression model posits that cancer cells spread to the metastatic site at an early stage, allowing metastasis to be detected before the primary site. In the second scenario, the tumor microenvironment selectively enhances tumor cell outgrowth at the metastatic site but inhibits growth at the primary site. This theory is supported by the existence of a clonal relationship between cells at the primary and metastatic sites [4].

Despite few CUP cases being diagnosed among all cancer subtypes, its unmet needs include precise identification of the primary organ of cancer origin, resulting in difficulty in appropriate treatment selection, thereby leading to an unfavorable survival outcome. Data retrieved from the Prince of Songkla University Hospital database revealed that only 12.9% of patients diagnosed with CUP received Medical Oncology services over the past 21 years (January 2002 to December 2022), reflecting a gap in CUP referral and underscoring the need for greater awareness of CUP and better recognition of the condition through a multidisciplinary approach.

Immunohistochemistry (IHC) is widely used as a standard pathological procedure for cancer diagnosis, prognosis, and prediction of treatment responses [13]. It is an essential ancillary technique in clinical and anatomical pathology because of its high reproducibility and reliability [14]. However, IHC alone cannot be used as an algorithm for diagnosing CUP. Novel molecular profiling procedures have been developed to identify the organ origin of CUP. The accuracy of GEP or comprehensive genomic profiling in identifying the organ origin of CUP ranges from 54% to 100%. Handorf et al. compared the diagnostic accuracy of GEP with that of IHC to identify the primary site of metastatic tumors using metastatic samples with known primary tumor sites. They found that GEP is more accurately identifiable in 89% of primary tumor sites compared with 83% of IHC sites (*p* = 0.023). The advantages of GEP have been predominantly observed in poorly differentiated and undifferentiated carcinomas. The underlying mechanism is the loss of some or all morphological features, which pathologists often use to identify possible primary organ origins in such tumors [15].

Another study by Zhen et al. used the expression of 90 genes in patients with multiple primary malignant tumors, defined as two or more histological malignancies in a single individual. GEP was used to classify organ origins and histology. They found that overall accuracy ranges from 87.5% to 95%, depending on tumor differentiation (well-, moderate-, or poorly differentiated tumors, squamous cell carcinoma) [16]. However, most studies have not used CUP specimens. Instead, they used specimens with known primary sites, which may limit the accuracy of determining organ origins in real-world practice. Integration of gene expression analysis into the diagnosis of CUP, in addition to conventional IHC, should be considered to achieve a more precise diagnosis and treatment. Further gene expression studies on CUP are needed to enhance diagnostic accuracy and improve treatment outcomes in these patients.

This study used the TCGA database to access RNA sequencing data from patients with colon cancer as a model to study the primary origin of CUP. The major advantage was that we could study molecular events in RNA and DNA across multiple cancer types using publicly available data. The standard RNA sequencing pipeline, when compared with the protein reference databases used for research, has not yet been fully completed, and data sharing in usable formats is lacking [17]. Therefore, we were interested in reanalyzing the RNA sequencing data. However, there are some incomplete annotations of patient outcomes and associated clinical data and metadata with relatively short clinical follow-up intervals [18,19].

We identified three upregulated genes in the lymph node and liver metastasis groups: *ROPN1*, *Y_RNA*, and *OR4F4*. *ROPN1* is a sperm-binding protein that interacts with Rho GTPase and is involved in sperm motility [20]. A previous study of *ROPN1* in melanoma found that it is expressed in melanoma cell lines [21]. Another study on triple-negative breast cancer (TNBC) revealed that *ROPN1* is upregulated in a TNBC dataset, cell lines, and human tissue using IHC. Moreover, *ROPN1* promotes cell migration and metastasis in vitro and in vivo, respectively. They also reported that upregulated *ROPN1* is associated with poor prognosis in patients with TNBC [22]. Villacis et al. evaluated patients with hereditary cancer syndrome or familial aggregation of cancer to investigate germline and copy number variants using whole-exome sequencing. They found *ROPN1* variants in all individuals from one family with colon, breast, and endometrial cancers, which were likely pathological variants [23]. While *ROPN1* dysregulation is observed in colon cancer, elevated expression is also detected in subsets of other malignancies (e.g., melanoma, breast, and gynecologic carcinomas). This cross-cancer expression pattern demonstrates that *ROPN1* is not exclusive to colon cancer lineage; therefore, further analysis of this mechanism is required in colon cancer and metastasis.

Ribonucleoprotein particles are complex molecules that include both proteins and RNAs. *Y_RNA*, a small non-coding RNA, represents the RNA component of the 60 kDa protein (Ro60) ribonucleoprotein particles [24]. This gene is involved in chromosomal DNA replication [25]. *Y_RNA* expression is upregulated in bladder, cervical, colon, kidney, lung, and prostate tumors compared with that in normal tissue [26]. Thus, *Y_RNA* upregulation may reflect generalized oncogenic activity rather than tissue-lineage specificity. Further analysis across paired primary/metastatic panels from multiple cancer types is mandatory before evaluating any lineage-tracing utility of *Y_RNA*. However, another upregulated gene, *OR4F4*, has not been widely studied. While our findings demonstrated significant dysregulation of *ROPN1*, *Y_RNA*, and *OR4F4* in the colon cancer lymph node and liver metastasis groups, these biomarkers should be interpreted cautiously as exploratory metastasis-associated genes rather than lineage-specific diagnostics.

Our study has some limitations. First, we retrieved a limited amount of available RNA sequencing-based data from both primary and metastatic sites. The major reason for this limitation is that metastatic tissues are usually not needed for cancer diagnosis or treatment, resulting in a low volume of archival tissues in the database. We are aware of the possibility of false-positive DGE caused by a small sample size, and encourage expanding the metastatic tissue cohort in future studies. Second, we faced the challenge of selecting a common cancer with retrievable genomic data as a prototype for a comparative study. This study used colon cancer as a model; however, the revealed DGE might not reflect organ specificity or diagnostic utility for CUP. Therefore, as a prospective suggestion for future research, metastatic tissues with adequate clinical data and comprehensive genomic profiles should be used to achieve greater accuracy in CUP diagnosis and improve treatment outcomes.

## 5. Conclusions

Systematic analysis and bioinformatics-based approaches are potentially useful methods for identifying exploratory metastasis biomarkers in patients with CUP. Further evaluation across paired primary/metastatic panels from diverse cancer types is required to assess the lineage-tracing potential in CUP. The small fraction of patients receiving Medical Oncology services highlights the need for greater awareness of this cancer subtype to improve recognition and management. Reanalysis of gene expression data is an approach for narrowing down candidate and target genes of interest. More widely used RNA sequencing data and accurate protocols will enable researchers to better compare platforms and experiments. Comprehensive genomic profiling has been implemented to enhance primary organ prediction in CUP, in addition to conventional pathological information derived from IHC.

## Figures and Tables

**Table 1:** Patients with cancer of unknown primary at the Prince of Songkla University Hospital from January 2002 to December 2022.

CUP in Years, n (%)	2002–2005	2006–2009	2010–2013	2014–2017	2018–2022	*p*-Value
Patients (n = 1193)	226 (18.9)	277 (23.2)	216 (18.1)	214 (17.9)	260 (21.8)	
Medical Oncology Service (n = 154)	11 (7.1)	22 (14.3)	33 (21.4)	34 (22.1)	54 (35.1)	<0.001
Non-Medical Oncology Service (n = 1039)	215 (20.7)	255 (24.6)	183 (17.6)	180 (17.3)	206 (19.8)	<0.001
*p*-value	<0.001	0.007	0.301	0.186	<0.001	

CUP, cancer of unknown primary.

## Data Availability

The data supporting the findings of this study are openly available through TCGA and the HCMI-CMDC project (https://portal.gdc.cancer.gov/projects/HCMI-CMDC (accessed on 6 November 2023)). The datasets generated and/or analyzed in the current study are available from the corresponding author upon reasonable request.

## Supplementary Materials

The following supporting information can be downloaded at https://www.mdpi.com/article/10.3390/cimb48101058/s1. Refs. [27,28,29,30] are in the Supplementary Materials (Table S2).

## Abbreviations

The following abbreviations are used in this manuscript:

CUP	Cancer of unknown primary
DGE	Differential gene expression
FDR	False discovery rate
FC/log2FC	Fold change/log2 fold change
GEPIA	Gene Expression Profiling Interactive Analysis
GEP	Gene expression profiling
HR	Hazard ratio
IHC	Immunohistochemistry
PSU	Prince of Songkla University
TCGA	The Cancer Genome Atlas
TNBC	Triple-negative breast cancer
TPM	Transcripts per million

## References

[1] Kolling S., Ventre F., Geuna E., Milan M., Pisacane A., Boccaccio C., Sapino A., Montemurro F. (2019). “Metastatic cancer of unknown primary” or “primary metastatic cancer”?. Front. Oncol..

[2] Laprovitera N., Riefolo M., Ambrosini E., Klec C., Pichler M., Ferracin M. (2021). Cancer of unknown primary: Challenges and progress in clinical management. Cancers.

[3] Kato S., Alsafar A., Walavalkar V., Hainsworth J., Kurzrock R. (2021). Cancer of unknown primary in the molecular era. Trends Cancer.

[4] Rassy E., Assi T., Pavlidis N. (2020). Exploring the biological hallmarks of cancer of unknown primary: Where do we stand today?. Br. J. Cancer.

[5] Losa F., Fernández I., Etxaniz O., Giménez A., Gomila P., Iglesias L., Longo F., Nogales E., Sánchez A., Soler G. (2022). SEOM-GECOD clinical guideline for unknown primary cancer (2021). Clin. Transl. Oncol..

[6] Akhmedov M., Martinelli A., Geiger R., Kwee I. (2020). Omics Playground: A comprehensive self-service platform for visualization, analytics and exploration of Big Omics Data. NAR Genom. Bioinform..

[7] Hu S.P., Ge M.X., Gao L., Jiang M., Hu K.W. (2021). LncRNA HCP5 as a potential therapeutic target and prognostic biomarker for various cancers: A meta-analysis and bioinformatics analysis. Cancer Cell Int..

[8] Colaprico A., Silva T.C., Olsen C., Garofano L., Cava C., Garolini D., Sabedot T.S., Malta T.M., Pagnotta S.M., Castiglioni I. (2016). TCGAbiolinks: An R/Bioconductor package for integrative analysis of TCGA data. Nucleic Acids Res..

[9] Silva T.C., Colaprico A., Olsen C., D’Angelo F., Bontempi G., Ceccarelli M., Noushmehr H. (2016). TCGA workflow: Analyze cancer genomics and epigenomics data using Bioconductor packages. F1000Research.

[10] Mounir M., Lucchetta M., Silva T.C., Olsen C., Bontempi G., Chen X., Noushmehr H., Colaprico A., Papaleo E. (2019). New functionalities in the TCGAbiolinks package for the study and integration of cancer data from GDC and GTEx. PLoS Comput. Biol..

[11] Chen Y., Lun A.T.L., Smyth G.K. (2016). From reads to genes to pathways: Differential expression analysis of RNA-Seq experiments using Rsubread and the edgeR quasi-likelihood pipeline. F1000Research.

[12] Tang Z., Li C., Kang B., Gao G., Li C., Zhang Z. (2017). GEPIA: A web server for cancer and normal gene expression profiling and interactive analyses. Nucleic Acids Res..

[13] Duraiyan J., Govindarajan R., Kaliyappan K., Palanisamy M. (2012). Applications of immunohistochemistry. J. Pharm. Bioallied Sci..

[14] Lin F., Chen Z. (2014). Standardization of diagnostic immunohistochemistry: Literature review and Geisinger experience. Arch. Pathol. Lab. Med..

[15] Handorf C.R., Kulkarni A., Grenert J.P., Weiss L.M., Rogers W.M., Kim O.S., Monzon F.A., Halks-Miller M., Anderson G.G., Walker M.G. (2013). A multicenter study directly comparing the diagnostic accuracy of gene expression profiling and immunohistochemistry for primary site identification in metastatic tumors. Am. J. Surg. Pathol..

[16] Zheng Y., Sun Y., Kuai Y., Fu G., An H., Chen J., Chen J., Zhu J., Wo Y., Wu Y. (2021). Gene expression profiling for the diagnosis of multiple primary malignant tumors. Cancer Cell Int..

[17] Shome M., MacKenzie T.M.G., Subbareddy S.R., Snyder M.P. (2024). The importance, challenges, and possible solutions for sharing proteomics data while safeguarding individuals’ privacy. Mol. Cell. Proteom..

[18] Liu J., Lichtenberg T., Hoadley K.A., Poisson L.M., Lazar A.J., Cherniack A.D., Kovatich A.J., Benz C.C., Levine D.A., Lee A.V. (2018). An integrated TCGA pan-cancer clinical data resource to drive high-quality survival outcome analytics. Cell.

[19] Wang X., Liu Q., Zhang B. (2014). Leveraging the complementary nature of RNA-Seq and shotgun proteomics data. Proteomics.

[20] Fujita A., Nakamura K., Kato T., Watanabe N., Ishizaki T., Kimura K., Mizoguchi A., Narumiya S. (2000). Ropporin, a sperm-specific binding protein of rhophilin, that is localized in the fibrous sheath of sperm flagella. J. Cell Sci..

[21] Da Gama Duarte J., Woods K., Quigley L.T., Deceneux C., Tutuka C., Witkowski T., Ostrouska S., Hudson C., Tsao S.C.H., Pasam A. (2021). Ropporin-1 and 1B are widely expressed in human melanoma and evoke strong humoral immune responses. Cancers.

[22] Liu Q., Huang X., Li Q., He L., Li S., Chen X., Ouyang Y., Wang X., Lin C. (2020). Rhophilin-associated tail protein 1 promotes migration and metastasis in triple negative breast cancer via activation of RhoA. FASEB J..

[23] Villacis R.A.R., Côrtes L., Basso T.R., do Canto L.M., Souza J.S., Aagaard M.M., da Cruz Formiga M.N., Aguiar S., Achatz M.I., Rogatto S.R. (2024). Germline DNA damage repair gene alterations in patients with metachronous breast and colorectal cancer. Int. J. Mol. Sci..

[24] Gulìa C., Signore F., Gaffi M., Gigli S., Votino R., Nucciotti R., Bertacca L., Zaami S., Baffa A., Santini E. (2020). Y RNA: An overview of their role as potential biomarkers and molecular targets in human cancers. Cancers.

[25] Christov C.P., Gardiner T.J., Szüts D., Krude T. (2006). Functional requirement of noncoding Y RNAs for human chromosomal DNA replication. Mol. Cell. Biol..

[26] Christov C.P., Trivier E., Krude T. (2008). Noncoding human Y RNAs are overexpressed in tumours and required for cell proliferation. Br. J. Cancer.

[27] Kim E.K., Song M.J., Jung Y., Lee W.S., Jang H.H. (2019). Proteomic analysis of primary colon cancer and synchronous solitary liver metastasis. Cancer Genom. Proteom..

[28] Smith J.J., Deane N.G., Wu F., Merchant N.B., Zhang B., Jiang A., Lu P., Johnson J.C., Schmidt C., Bailey C.E. (2010). Experimentally derived metastasis gene expression profile predicts recurrence and death in patients with colon cancer. Gastroenterology.

[29] Levine E.A., Blazer D.G., Kim M.K., Shen P., Stewart J.H., Guy C., Hsu D.S. (2012). Gene expression profiling of peritoneal metastases from appendiceal and colon cancer demonstrates unique biologic signatures and predicts patient outcomes. J. Am. Coll. Surg..

[30] Sargent D.J., Shi Q., Gill S., Louvet C., Everson R.B., Kellner U., Clancy T.E., Pipas J.M., Resnick M.B., Meyers M.O. (2014). Molecular testing for lymph node metastases as a determinant of colon cancer recurrence: Results from a retrospective multicenter study. Clin. Cancer Res..

