# Supplementary material for: Determination of Exploratory Metastasis Biomarkers in Cancer of Unknown Primary Using Metastatic Colon Cancer as a Model: RNA Sequencing Bioinformatics Analysis
Source: Curr Issues Mol Biol. 2026 Oct 14;48(10):1058. doi: 10.3390/cimb48101058 (PMC13703600; doi:10.3390/cimb48101058)
Supplement: Supplementary file 1 [file cimb-48-01058-s001.zip › cimb-4528943-supplementary.pdf]

Supplementary Materials

Determination of exploratory metastasis biomarkers in cancer of unknown primary using metastatic colon cancer as a model: RNA sequencing bioinformatics analysis

Rawikant Kamolphiwong[1], Pongsakorn Choochuen[2], Maliwan Songserm[1], Patcharaporn Thongwatchara[1], Arunee Dechaphunkul[1], Chirawadee Sathitruangsak[1], Jirapat Wonglhow[1], Patrapim Sunpaweravong[1]

[1]Division of Medical Oncology, Department of Internal Medicine, Faculty of Medicine, Prince of Songkla University, Songkhla 90110, Thailand

[2]Department of Biomedical Sciences and Biomedical Engineering, Faculty of Medicine, Prince of Songkla University, Songkhla 90110, Thailand

Corresponding author: Patrapim Sunpaweravong, M.D., Ph.D.

E-mail: spatrapi@medicine.psu.ac.th

ORCID: 0000-0001-9787-270X

Table S1 Yearly total number of patients with cancer of unknown primary in Prince of Songkla University Hospital and those who received Medical Oncology services

Year	All patients with CUP (N)	Patients with CUP and Medical Oncology services
2002	51	0
2003	46	0
2004	58	5
2005	71	6
2006	71	5
2007	76	5
2008	61	6
2009	69	6
2010	58	7
2011	50	6
2012	53	9
2013	55	11
2014	52	8
2015	57	4
2016	51	11
2017	54	11
2018	58	15
2019	63	16
2020	57	7
2021	36	4
2022	46	12
Total	**1193**	**154**

CUP, cancer of unknown primary.

Table S2 Articles on colon cancer with gene expression

Title	Comparison	Reported genes
Proteomic Analysis of Primary Colon Cancer and Synchronous Solitary Liver Metastasis [27]	Liver metastatic site VS colon site	Examples: *CAPNS1 SERINA1 APOA1 COTL1 ACTG1 ACTC1 ITLN1 ATP5B PDIA5 DES S100A9 PSMB3 GLOD4 PDHB DDAH1 CKB UQCRC1 ANXA6 SOD1 ARPC5 CAPZB DDAH2 ACTR3 GSN PARK7 DBI*
Experimentally derived metastasis gene expression profile predicts recurrence and death in patients with colon cancer [28]	Concordance gene between metastatic colon patients and metastatic colon cell lines in vivo	*CXCR7 AK1 ACTB MGP HES1 TMEM14A EGR1 VDR C6orf64 NQO1 STOX2 ACYP2 SPRY4 DCTD TACC2 PDLIM5*
Gene expression profiling of peritoneal metastases from appendiceal and colon cancer demonstrates unique biologic signatures and predicts patient outcomes [29]	Peritoneal appendiceal and peritoneal colorectal gene clustering	No report
Molecular testing for lymph node metastases as a determinant of colon cancer recurrence: results from a retrospective multicenter study [30]	Guanylyl cyclase C (*GCC*) mRNA levels in lymph nodes on colon cancer recurrence	Guanylyl cyclase C (*GCC*)

VS, versus.

Table S3 RNA gene expression database retrieved from TCGA

Patient No.	Case submitter ID	Case UUID (GDC accession)	Sample submitter ID	Sample UUID (GDC accession)	Tissue Bx	Analysis group	Site of resection or biopsy	Tissue or organ of origin	Project
1	HCM-CSHL-0056-C18	bfe15f44-e1dd-46ed-b429-908822d0a781	HCM-CSHL-0056-C18-01B	42994984-d406-4c83-9df4-7b461b118143	Primary	Primary	Cecum	Colon, NOS	HCMI-CMDC (GDC)
2	HCM-CSHL-0057-C18	d000dcfe-181c-46fe-93ba-5fce8336b52d	HCM-CSHL-0057-C18-01B	424c78de-d4fa-4cfb-a39c-b4dd8b94a3a9	Primary	Primary	Sigmoid colon	Colon, NOS	HCMI-CMDC (GDC)
3	HCM-CSHL-0060-C18	3b797720-33ef-45e0-9830-5f6007f1d5a7	HCM-CSHL-0060-C18-01B	7bda59a9-087a-42b0-bea8-c5eedaf68042	Primary	Primary	Sigmoid colon	Colon, NOS	HCMI-CMDC (GDC)
4	HCM-CSHL-0061-C18	118bf677-caed-4f7f-a837-dd966dce5ca7	HCM-CSHL-0061-C18-01A	0ca75f2e-b1e8-4f74-acda-7e390d133064	Primary	Primary	Sigmoid colon	Colon, NOS	HCMI-CMDC (GDC)
5	HCM-CSHL-0063-C18	e802e579-5293-465c-a867-74e290268299	HCM-CSHL-0063-C18-01A	d002a9f5-d26c-4974-aa4d-6fdfbb77833a	Primary	Primary	Sigmoid colon	Colon, NOS	HCMI-CMDC (GDC)
6	HCM-CSHL-0064-C18	cd6f37d5-d192-48b9-a63f-54d8d1e810c6	HCM-CSHL-0064-C18-01A	cfae2bc8-5218-4fa2-a31f-2e8bb99f36d0	Primary	Primary	Sigmoid colon	Colon, NOS	HCMI-CMDC (GDC)
8	HCM-CSHL-0141-C18	23443700-fe39-4fd5-862f-ffb02cda3947	HCM-CSHL-0141-C18-01A	b1fcca94-7577-4472-8a81-6a53818bce5b	Primary	Primary	Sigmoid colon	Colon, NOS	HCMI-CMDC (GDC)
9	HCM-CSHL-0142-C18	fdfd5770-4b64-4d35-b472-9c7908210b22	HCM-CSHL-0142-C18-01A	b58b98ee-0f2c-4a8b-bfd8-79d94301a8bb	Primary	Primary	Sigmoid colon	Colon, NOS	HCMI-CMDC (GDC)
10	HCM-CSHL-0160-C18	06d42c21-4c97-4171-ac00-3979a51a59d9	HCM-CSHL-0160-C18-01B	157e37cf-cda4-4936-ba0f-717f3349faa3	Primary	Primary	Ascending colon	Colon, NOS	HCMI-CMDC (GDC)
12	HCM-CSHL-0237-C18	c446e9cd-f5cc-4daa-b573-b261d7ea8f42	HCM-CSHL-0237-C18-01B	54eb924b-d15c-425a-ad3c-33344f577dd6	Primary	Primary	Sigmoid colon	Colon, NOS	HCMI-CMDC (GDC)
13	HCM-CSHL-0238-C18	67e3c17e-e524-4681-8162-99742c802256	HCM-CSHL-0238-C18-01B	bd8cc933-1318-4aa9-80e3-c5bcd1cd9fd7	Primary	Primary	Cecum	Colon, NOS	HCMI-CMDC (GDC)
14	HCM-CSHL-0239-C18	9ee323b8-6447-4447-b70f-a7260b3b8f8e	HCM-CSHL-0239-C18-01A	afbc1302-e19e-4271-9765-8dc053ce5cdc	Primary	Primary	Sigmoid colon	Colon, NOS	HCMI-CMDC (GDC)
15	HCM-CSHL-0241-C18	f9d5bd26-816b-4aaa-809f-19d4b8d74692	HCM-CSHL-0241-C18-01A	640e928c-bd78-4b02-885e-9f25d1260aff	Primary	Primary	Splenic flexure of colon	Colon, NOS	HCMI-CMDC (GDC)
15	HCM-CSHL-0241-C18	f9d5bd26-816b-4aaa-809f-19d4b8d74692	HCM-CSHL-0241-C18-85A	b49e71b5-3cda-40b9-a8a5-aed43ab4a4f6	Primary	Primary	Splenic flexure of colon	Colon, NOS	HCMI-CMDC (GDC)
17	HCM-CSHL-0247-C18	7fa8bc57-4bc7-48a5-99fe-98ed02fa17e8	HCM-CSHL-0247-C18-01A	ccf33afa-78f2-4d69-97a7-7fc8c4defa84	Primary	Primary	Cecum	Colon, NOS	HCMI-CMDC (GDC)
17	HCM-CSHL-0247-C18	7fa8bc57-4bc7-48a5-99fe-98ed02fa17e8	HCM-CSHL-0247-C18-85A	98bce62e-db3e-4b46-9161-0028bb94e0b3	Primary	Primary	Cecum	Colon, NOS	HCMI-CMDC (GDC)
18	HCM-CSHL-0257-C18	f0c6e5b9-3d64-456e-85ab-9978a91ffd43	HCM-CSHL-0257-C18-85A	d2b3827d-ec38-4fae-91da-7422b0c7d25b	Primary	Primary	Sigmoid colon	Colon, NOS	HCMI-CMDC (GDC)
19	HCM-CSHL-0258-C18	d77ca75a-491a-4616-9a1c-7b69fdd03585	HCM-CSHL-0258-C18-01A	94ee3758-87a5-4413-b130-c9ac57b67bde	Primary	Primary	Sigmoid colon	Colon, NOS	HCMI-CMDC (GDC)
19	HCM-CSHL-0258-C18	d77ca75a-491a-4616-9a1c-7b69fdd03585	HCM-CSHL-0258-C18-85D	17939a13-b24e-4e7a-9c14-715b63f7ec30	Primary	Primary	Sigmoid colon	Colon, NOS	HCMI-CMDC (GDC)
20	HCM-CSHL-0317-C18	de19bdf6-63b3-4e75-bf11-a7b6ebcea466	HCM-CSHL-0317-C18-85A	f46e8094-20ea-4cd9-a950-68c5bb008534	Primary	Primary	Ascending colon	Colon, NOS	HCMI-CMDC (GDC)
21	HCM-CSHL-0382-C19	7ee0c283-701b-47ac-bc3a-30f45e5c4eb2	HCM-CSHL-0382-C19-01A	c6d01326-dd06-4a25-ad78-61a4978ba775	Primary	Primary	Rectosigmoid junction	Colon, NOS	HCMI-CMDC (GDC)
21	HCM-CSHL-0382-C19	7ee0c283-701b-47ac-bc3a-30f45e5c4eb2	HCM-CSHL-0382-C19-85A	e6a4a148-dd54-4e83-9497-eabd314b70e0	Primary	Primary	Rectosigmoid junction	Colon, NOS	HCMI-CMDC (GDC)
22	HCM-CSHL-0426-C18	93eb458f-2b57-498b-a356-9b5540cc545c	HCM-CSHL-0426-C18-85A	6762c326-9637-4949-ae9f-84ed287c9193	Primary	Primary	Cecum	Colon, NOS	HCMI-CMDC (GDC)
22	HCM-CSHL-0426-C18	93eb458f-2b57-498b-a356-9b5540cc545c	HCM-CSHL-0426-C18-86A	4694330c-dd00-47bc-894c-d18bb91bc5e4	Primary	Primary	Cecum	Colon, NOS	HCMI-CMDC (GDC)
23	HCM-CSHL-0727-C18	28f77d34-e59d-4b80-8885-88d11c2ced74	HCM-CSHL-0727-C18-06A	b9921764-534c-4575-81a5-b06625f9f711	Primary	Primary	Rectosigmoid junction	Colon, NOS	HCMI-CMDC (GDC)
23	HCM-CSHL-0727-C18	28f77d34-e59d-4b80-8885-88d11c2ced74	HCM-CSHL-0727-C18-85M	6dca3041-58a6-4dcc-8f7d-16312d918374	Primary	Primary	Rectosigmoid junction	Colon, NOS	HCMI-CMDC (GDC)
24	HCM-CSHL-0729-C18	f6a13511-0819-4219-bc4b-6cb146629f0e	HCM-CSHL-0729-C18-01A	56499e3a-913e-4643-9799-c6e74e89749f	Primary	Primary	Ascending colon	Colon, NOS	HCMI-CMDC (GDC)
26	HCM-SANG-0268-C18	07a067d0-7dfc-4817-b4c5-9200da20a59f	HCM-SANG-0268-C18-01A-01D-A80T-32	6a530ae1-79fa-4536-bbf5-86b14a80563e	Primary	Primary	Transverse colon	Colon, NOS	HCMI-CMDC (GDC)
27	HCM-SANG-0269-C18	daf1dd34-2188-4cb0-95a5-286e0a0ba5ad	HCM-SANG-0269-C18-01A-01D-A80T-32	562e2579-23b5-42b0-be7f-0319e03f02c3	Primary	Primary	Sigmoid colon	Colon, NOS	HCMI-CMDC (GDC)
28	HCM-SANG-0273-C18	b3b23c0b-a9bd-434a-9875-5cfba04cfe7f	HCM-SANG-0273-C18-01A-01D-A80T-32	2c0d1e7a-d1d3-4ba5-ad08-ad45d60b90da	Primary	Primary	Ascending colon	Colon, NOS	HCMI-CMDC (GDC)
29	HCM-SANG-0274-C18	c7a011a7-e72c-4535-8367-43d07d80fb67	HCM-SANG-0274-C18-01A-01D-A80T-32	bccfe398-88b9-4a4f-afa7-aa905f1f2fd6	Primary	Primary	Transverse colon	Colon, NOS	HCMI-CMDC (GDC)
30	HCM-SANG-0281-C18	a13f7c10-76c8-473a-b87b-8b680706d189	HCM-SANG-0281-C18-01A-01D-A80T-32	903fd4de-bba7-456c-9942-29bae75486b0	Primary	Primary			HCMI-CMDC (GDC)
31	HCM-SANG-0282-C18	cf3e235e-676c-45de-a746-2ea45fd2142f	HCM-SANG-0282-C18-01A-01D-A80T-32	4c86f3ea-e307-42b2-b40a-345fef0a3cd4	Primary	Primary	Ascending colon	Colon, NOS	HCMI-CMDC (GDC)
32	HCM-SANG-0283-C18	eb537fd2-ac06-4f63-8c0a-0ca73dd06a3a	HCM-SANG-0283-C18-01A-01D-A80T-32	e6201481-cb5e-48db-afdc-3ed290edd2a6	Primary	Primary	Sigmoid colon	Colon, NOS	HCMI-CMDC (GDC)
33	HCM-SANG-0284-C18	96dea2e6-e29b-4e09-b708-ae3b010a9906	HCM-SANG-0284-C18-01A-01D-A80T-32	dcd6e8db-046e-4811-acb3-f85ad548e8f7	Primary	Primary	Sigmoid colon	Colon, NOS	HCMI-CMDC (GDC)
34	HCM-WCMC-0504-C18	031b40e7-2e1d-43e9-83a1-a4fae1e1e6a4	HCM-WCMC-0504-C18-01A	8cac4f4f-c223-4206-99f1-cfcbbfadd2cd	Primary	Primary	Transverse colon	Colon, NOS	HCMI-CMDC (GDC)
35	HCM-WCMC-0671-C18	603035e9-eef7-490a-a102-af4eb005fb73	HCM-WCMC-0671-C18-01A	063d463f-35fb-4648-81d1-2234a77270d1	Primary	Primary	Sigmoid colon	Colon, NOS	HCMI-CMDC (GDC)
16	HCM-CSHL-0245-C18	df69a9b0-63a6-47f4-b3a7-beb37edf100d	HCM-CSHL-0245-C18-85A	84cb8ace-99bf-43f8-90e6-928714974497	Primary	Lymph node	Lymph node, NOS	Colon, NOS	HCMI-CMDC (GDC)
16	HCM-CSHL-0245-C18	df69a9b0-63a6-47f4-b3a7-beb37edf100d	HCM-CSHL-0245-C18-85M	3893bc3e-c85d-4fa1-bb4e-4706c4881c7e	Metastasis	Lymph node	Lymph node, NOS	Colon, NOS	HCMI-CMDC (GDC)
17	HCM-CSHL-0247-C18	7fa8bc57-4bc7-48a5-99fe-98ed02fa17e8	HCM-CSHL-0247-C18-06A	ea572081-f101-48fa-a087-93b7c6638da6	Metastasis	Lymph node	Lymph node, NOS	Colon, NOS	HCMI-CMDC (GDC)
17	HCM-CSHL-0247-C18	7fa8bc57-4bc7-48a5-99fe-98ed02fa17e8	HCM-CSHL-0247-C18-85M	d6800827-a747-4c83-839f-73d8d581ce35	Metastasis	Lymph node	Lymph node, NOS	Colon, NOS	HCMI-CMDC (GDC)
7	HCM-CSHL-0065-C20	b5fe3fa5-0f46-4e63-8e1f-dc3d28734936	HCM-CSHL-0065-C20-06A	12d9056f-ed11-4b8c-8c06-8c3e1b7f3370	Metastasis	Liver	Liver	Colon, NOS	HCMI-CMDC (GDC)
11	HCM-CSHL-0164-C20	6058fb8d-eb5d-4dbc-b939-7e92c7cd2776	HCM-CSHL-0164-C20-06A	02e6c9a4-a7b8-4d03-bc61-5ee8c3b6ebd1	Metastasis	Liver	Liver	Colon, NOS	HCMI-CMDC (GDC)
11	HCM-CSHL-0164-C20	6058fb8d-eb5d-4dbc-b939-7e92c7cd2776	HCM-CSHL-0164-C20-85A	8b456564-310c-4393-b4c4-184e4c90be7e	Metastasis	Liver	Liver	Colon, NOS	HCMI-CMDC (GDC)
21	HCM-CSHL-0382-C19	7ee0c283-701b-47ac-bc3a-30f45e5c4eb2	HCM-CSHL-0382-C19-85N	0ecf3052-2d2e-41d0-8ba5-b3e1b83e9897	Metastasis	Liver	Liver	Colon, NOS	HCMI-CMDC (GDC)
25	HCM-SANG-0265-C18	f8a9aab0-6017-483d-b053-9393c6a5af2d	HCM-SANG-0265-C18-06A	b38ea180-86e6-4868-a124-3b18edfe201b	Metastasis	Liver	Liver	Colon, NOS	HCMI-CMDC (GDC)

TCGA, The Cancer Genome Atlas.

References

27. Kim E.K.; Song M.J.; Jung Y.; Lee W.S.; Jang H.H. Proteomic analysis of primary colon cancer and synchronous solitary liver metastasis. *Cancer Genomics Proteomics* **2019**, *16*, 583–592. Available online: https://doi [https://doi.org/10.21873/cgp.20161].

28. Smith J.J.; Deane N.G.; Wu F.; Merchant N.B.; Zhang B.; Jiang A.; Lu P.; Johnson J.C.; Schmidt C.; Bailey C.E.; et al. Experimentally derived metastasis gene expression profile predicts recurrence and death in patients with colon cancer. *Gastroenterology* **2010**, *138*, 958–968. Available online: https://doi [https://doi.org/10.1053/j.gastro.2009.11.005].

29. Levine E.A.; Blazer 3rd D.G.; Kim M.K.; Shen P.; Stewart J.H.; Guy C.; Hsu D.S. Gene expression profiling of peritoneal metastases from appendiceal and colon cancer demonstrates unique biologic signatures and predicts patient outcomes. *J. Am. Coll. Surg.* **2012**, *214*, 599–606; discussion 606 [https://doi.org/10.1016/j.jamcollsurg.2011.12.028].

30. Sargent D.J.; Shi Q.; Gill S.; Louvet C.; Everson R.B.; Kellner U.; Clancy T.E.; Pipas J.M.; Resnick M.B.; Meyers M.O.; et al. Molecular testing for lymph node metastases as a

determinant of colon cancer recurrence: results from a retrospective multicenter study. *Clin. Cancer Res.* **2014**, *20*, 4361–4369 [https://doi.org/10.1158/1078-0432.CCR-13-2659].
